# Assessing service availability and readiness to manage cervical cancer in Bangladesh

**DOI:** 10.1186/s12885-021-08387-2

**Published:** 2021-06-05

**Authors:** Shagoofa Rakhshanda, Koustuv Dalal, Hasina Akhter Chowdhury, Cinderella Akbar Mayaboti, Progga Paromita, A. K. M. Fazlur Rahman, A. H. M. Eanayet Hussain, Saidur Rahman Mashreky

**Affiliations:** 1 Centre for Injury Prevention and Research, Bangladesh (CIPRB), Dhaka, Bangladesh; 2 School of Health Sciences, Mid Sweden University, Sundsvall, Sweden; 3 School of Medicine and Health Care, al-Farabi Kazakh National University, Almaty, Kazakhstan; 4 Kirtipasha Health and Family Welfare Centre, Jhalokathi Sadar Upazilla, Bangladesh; 5 Department of Epidemiology, Bangladesh University of Health Science (BUHS), Dhaka, Bangladesh; 6 Directorate General of Medical Education, Dhaka, Bangladesh; 7 Department of Non-communicable Diseases, Bangladesh University of Health Science (BUHS), Dhaka, Bangladesh

**Keywords:** Bangladesh, Cervical cancer, Service availability and readiness assessment, WHO SARA

## Abstract

**Background:**

The second most common cancer among females in Bangladesh is cervical cancer. The national strategy for cervical cancer needs monitoring to ensure that patients have access to care. In order to provide accurate information to policymakers in Bangladesh and other low and middle income countries, it is vital to assess current service availability and readiness to manage cervical cancer at health facilities in Bangladesh.

**Methods:**

An interviewer-administered questionnaire adapted from the World Health Organization Service Availability and Readiness Assessment Standard Tool was used to collect cross-sectional data from health administrators of 323 health facilities in Bangladesh. Services provided were categorized into domains and service readiness was determined by mean readiness index (RI) scores. Data analysis was conducted using STATA version 13.

**Results:**

There were seven tertiary and specialized hospitals, 118 secondary level health facilities, 124 primary level health facilities, and 74 NGO/private hospitals included in the study. Twenty-six per cent of the health facilities provided services to cancer patients. Among the 34 tracer items used to assess cancer management capacity of health facilities, four cervical cancer-specific tracer items were used to determine service readiness for cervical cancer. On average, tertiary and specialized hospitals surpassed the readiness index cutoff of 70% with adequate staff and training (100%), equipment (100%), and diagnostic facilities (85.7%), indicating that they were ready to manage cervical cancer. The mean RI scores for the rest of the health facilities were below the cutoff value, meaning that they were not prepared to provide adequate cervical cancer services.

**Conclusion:**

The health facilities in Bangladesh (except for some tertiary hospitals) lack readiness in cervical cancer management in terms of guidelines on diagnosis and treatment, training of staff, and shortage of equipment. Given that cervical cancer accounts for more than one-fourth of all female cancers in Bangladesh, management of cervical cancer needs to be available at all levels of health facilities, with primary level facilities focusing on early diagnosis. It is recommended that appropriate standard operating procedures on cervical cancer be developed for each level of health facilities to contribute towards attaining sustainable developmental goals.

## Background

Over the years, the increasing burden of non-communicable diseases (NCDs), such as diabetes, hypertension, cardiovascular diseases, and cancer has become a significant global public health concern [1, 2]. Studies show that about 80% of deaths due to NCDs occur in low- and middle-income countries (LMICs) [3]. Poverty has a bi-directional relationship with NCDs, as the poorer people are at higher risk of NCDs and the associated high treatment costs increase the economic burden [4]. In 2018, 9.6 million global deaths were due to cancer, making it the second leading cause of death, of which approximately 70% occurred in LMICs [5, 6]. Only 26% of people in LMICs have access to diagnostic services for cancer [6]. Lung, colorectum and breast (in females) cancers are the most frequently occurring cancers globally [6].

The second most predominant type of cancer among women in LMICs is cervical cancer, next to breast cancer [7, 8]. Eighty-four per cent of new cases of cervical cancer and 85% of total deaths from cervical cancer occur among women in developing countries [7, 8]. Early diagnoses and treatment of cervical cancer is cost-effective and can improve health outcomes [7]. Human Papilloma Virus (HPV) has been identified as the causative agent responsible for cervical cancer. Though about 90% of HPV infection is clear within 2 years, it may persist and progress to cervical cancer in 15–20 years [9].

Like other LMICs, cervical cancer is the second most common cancer among females in Bangladesh (12% of female cancers), with 8068 new cases and 5214 deaths caused by it every year [10–13]. The existing cancer registry facility in Bangladesh is gradually being updated [14]. Through the National Cervical Cancer Control Program, the Government of Bangladesh (GOB) gradually developed cervical cancer screening facilities at all districts and Upazila levels – in about 400 centers. The screening method adopted was Visual Inspection of Cervix with Acetic Acid (VIA) for women 30 years and above [11]. However, the nationwide screening coverage of cervical cancer is less than 10% [14].

With scientific advancement and changing health dynamics, health systems researchers have identified critical areas that can improve cancer management in developing countries if addressed [15]. These include structural and functional strengthening, enabling follow-up, quality assurance, and evaluation and monitoring [15]. In line with these recommendations, the National Strategy for Cervical Cancer Prevention and Control Bangladesh (2017–2022) was developed, aiming to strengthen the ongoing National Cervical Cancer Control Program. The National Strategy includes introducing vaccination for adolescent girls against the Human Papillomavirus (HPV) through the existing Expanded Program on Immunization (EPI) and strengthening modalities for implementing population-based cervical cancer screening and necessary treatment through the current healthcare delivery system. The strategy envisions that the cervical cancer screening program will be implemented collaboratively by Primary Health Care (PHC), Reproductive Health, Non-communicable Disease Control (NCDC) program, Hospital Service Management (HSM) and Management Information System (MIS) of Directorate General of Health Services (DGHS) and Directorate General of Family Planning (DGFP). At the tertiary level, gynecologists and gynecologic oncologists are supposed to be available for surgical management of early-stage cancers, radiation therapy and chemotherapy facilities [11].

At this juncture of mid-way in the implementation of the National Strategy for Cervical Cancer Prevention and Control Bangladesh (2017–2022), an assessment of the program implementation using the Service Availability and Readiness Assessment (SARA) Survey may be of value to note the achievements, and highlight the areas where further strengthening or updates in line with current scientific research may be needed [16]. The study was undertaken with the expectation that the findings will feed into the mid-term assessment of the Government’s strategy to improve the management of cervical cancer. The study findings may also aware other LMICs to focus on updating their ongoing program on cervical cancer management at different levels of health service delivery.

## Methods

### Study design

It was a cross-sectional sub-study of a more extensive parent study called Service Availability and Readiness Assessment (SARA) Survey for NCDs and Disability Service Delivery System in Bangladesh. The study was conducted from December 2017 to June 2018. In this sub-study, variables for assessing the availability and readiness of cancer-related services (mainly focusing on cervical cancer services) were considered.

WHO SARA Standard Tool was used to collect data on the availability of medical equipment related to cancer including their location, functional status, and components of support systems (e.g., logistics, maintenance, and management) [16].

### Study sample and sampling technique

A list of all available public and private health facilities was compiled at the primary, secondary and tertiary levels in Bangladesh using the Management Information System (MIS) and and hospital administration records from the Directorate General of Health Services (DGHS); the National Institute of Population Research and Training (NIPORT) and from the Bangladesh Health Facility Survey (BHFS) 2014 [17, 18]. The list constituted of a total of 19,184 health facilities.

Of these 19,184 health facilities, the parent study of SARA Survey for NCDs and Disability Service Delivery System in Bangladesh selected a sample of 590 health facilities at various levels. These included ten randomly selected tertiary and specialized hospitals for NCDs (i.e., 10%). All secondary level health facilities, that is, 60 district hospitals (DHs) and 61 Mother and Child Welfare Centre (MCWCs), were also included in the sample. Among the primary level health facilities, the sample also included 128 (30% of all) Upazila Health Complexes (UHCs), and 256 Family Welfare Centres (FWCs) and Community Clinics (CCs). Further, we included randomly selected 75 NGOs/ private hospitals.

**Fig. 1:**
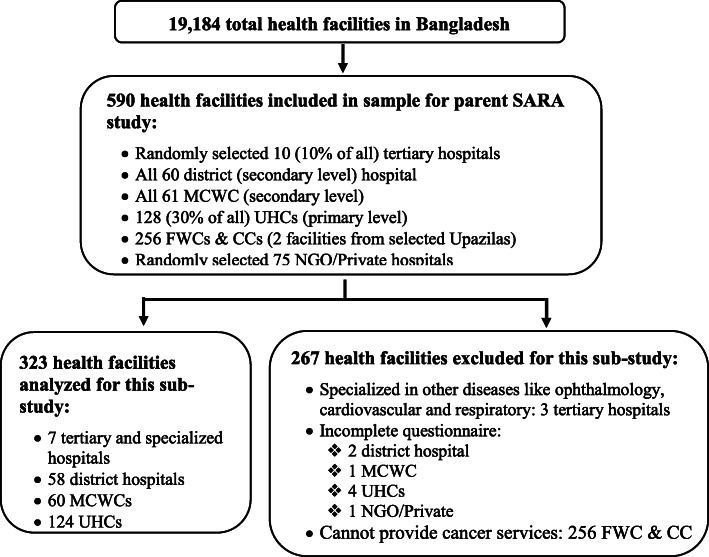
Sampling flow diagram

This sub-study then excluded 267 health facilities from analysis, which included specialized hospitals that did not focus on cervical cancer care (such as those devoted solely to ophthalmology) and facilities where a staff member was not available to complete the survey. The study included a final sample size of 323 health facilities, as depicted in Fig. 1.

The head of each hospital or a management staff member who would have sufficient knowledge of hospital capacity and operations were selected to participate in the survey (Table 1).

**Table 1:** Type of interviewees from different health facilities

Facility type	Interviewee type
Tertiary and Specialized hospital	Director or their representative
District hospital (DH) (Secondary level)	Civil Surgeon (CS)/Superintendent
Maternal and Child Welfare Centre (MCWC) (Secondary level)	Medical Officer
Upazila Heath Complex (UHC) (Primary level)	Union Health and Family Planning Officer (UHFPO) or Resident Medical Officer (RMO)
NGO / Private hospital	Head of NGO/private hospital

### Data collection

Forty-eight interviewers, all Bachelor of Medicine and Bachelor of Surgery (MBBS) graduates, were trained for 2 days on the data collection instrument and data entry into RedCap software using tablets. The interviewers received instructions for uploading the data immediately to the server from each health facility, enabling researchers at the head office to monitor the data collection in real-time. The health facilities included in this study were contacted beforehand and an appointment was set for the interviews. The interviews were conducted in-person and recorded.

### Statistical analysis

Descriptive analyses were performed based on the dataset using the standard core indicators (staff and guideline, equipment, diagnostic facilities, and medicines) and corresponding tracer indicators, as per the reference manual of SARA [19]. There was one variable for assessing the availability and 34 variables for assessing the readiness of cancer related services. Of these 34 variables, four were specific to cervical cancer, which was further analyzed for assessing readiness focusing specifically on services for patients with cervical cancer. Table 2 shows a list of tracer indicators. Data were compiled and analyzed by standard statistical procedures using STATA version 13.

**Table 2:** Tracer indicators in respective domains of cancer services

Domains	Tracer Items
Staff and guideline	Oncologists
	Palliative care specialist
	Trained Nurse
	Guideline ^a^
	Training ^a^
Equipment	Beds available for cancer patients
	Speculum ^a^
Diagnostic facility	Acetic Acid ^a^
	Sputum for Cytology
	Stool occult blood test (OBT)
	Cancer antigen 15.3 (CA 15.3)
	Cancer antigen 19.9 (CA 19.9)
	Carcinoembryonic antigen (CEA)
	Cancer antigen 125 (CA125)
	Alpha Keto Protein
	Endoscopy of upper gastrointestinal tract
	Colonoscopy
	Pituitary tumor-transforming gene 1-binding factor (PBF)
	Bone Marrow Study
	Biopsy for Histopathology
	Fine Needle Aspiration Cytology (FNAC)
Medicines	Hydrocortisone
	Pheniramine
	Ondanseron
	Haloperidol
	Morphin injection
	Morphin tablet
	Lorazepam
	Hyoscine butylebromide
	Broad Spectrum Antibiotics
	Chemotherapeutic drugs

^a^ cervical cancer specific tracer items

Two outcomes, the “availability” and “readiness” of cancer services, were calculated based upon previous studies that utilized the SARA tool [20, 21]. Service “availability” was assessed by asking if the health facility provided any service for patients with any cancer (yes or no), not specific to cervical cancer. This gave data on the availability of overall cancer services at different levels of health facilities. Service “readiness” was calculated as a composite score using 34 variables and consisted of five stages:

Data was collected on the availability of any type of cancer service readiness tracer indicators at all health facilities.

Tracer indicator scores were calculated for all cancer tracer indicators at each health facility level, using the following formula: number of health facilities with individual tracer indicators present *100/total number of health facilities.

Next, the tracer indicator scores specifically for cervical cancer were extracted (guideline, training, speculum, and acetic acid) which fell under three domains (staff and guideline, equipment, and diagnostic tool). Two of the indicators, guidelines and training, covered the domain of staff and training. Each of the other two indicators covered the domains of equipment and diagnostic tool respectively.

In the next step, the cervical cancer-specific readiness index (CSRI) scores for the cervical cancer-specific domains were calculated for each health facility level. CSRI was done by determining the mean of all tracer indicator scores for each domain (sum of tracer indicator score in each domain/number of tracer indicators in each domain) for each facility level.

The mean cervical cancer-specific readiness index (mean CSRI) score was calculated at each level of health facility by determining the average of the CSRI score of all three domains (sum of readiness index in each domain/number of domains) at each facility level.

Cervical cancer-specific readiness index (CSRI) scores and mean index scores were then compared to a cutoff level of 70%, as evident from a study in Zambia that also used the SARA tool and considered health facilities to be “ready” to manage NCDs when their mean readiness index scores were above the same cutoff score [21]. All analyses were performed using STATA version 13.

## Results

### Characteristics of health facilities

A total of 323 health facilities were assessed, where 248 (76.8%) were public and 75 (23.2%) were from the private sector. Facilities included 124 UHCs (primary level) (38.4%), 74 private/NGO hospitals (22.9%), 58 DH (secondary level) (18.0%), 60 MCWC (secondary level) (18.6%), and 7 tertiary level specialized hospitals (2.2%). Most of the NGO and private hospitals are secondary level health facilities. However, only one of the tertiary level specialized hospital was a private hospital. More than one-fourth of facilities were situated in the Dhaka division (Table 3).

**Table 3:** Type and distribution of health facilities (*n* = 323)

Characteristics	Category	Number (%) of health facilities
**Ownership**	Public	248 (76.8)
	Private	75 (23.2)
**Level of health facility**	Tertiary and Specialized hospital	7 (2.2)
	District hospital (Secondary)	58 (18.0)
	MCWC (Secondary)	60 (18.6)
	Upazila Heath Complex (Primary)	124 (38.4)
	NGO / Private hospital	74 (22.9)
**Division**	Dhaka	83 (25.7)
	Barishal	30 (9.3)
	Chittagong	52 (16.1)
	Khulna	51 (15.8)
	Rajshahi	43 (13.3)
	Rangpur	43 (13.3)
	Sylhet	21 (6.5)

### Service “availability” for overall cancer

**Fig. 2:**
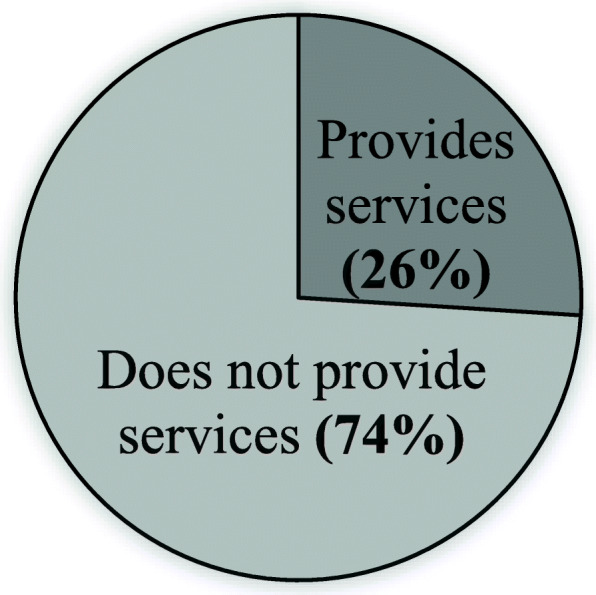
Proportion of cancer services providing health facilities

**Fig. 3:**
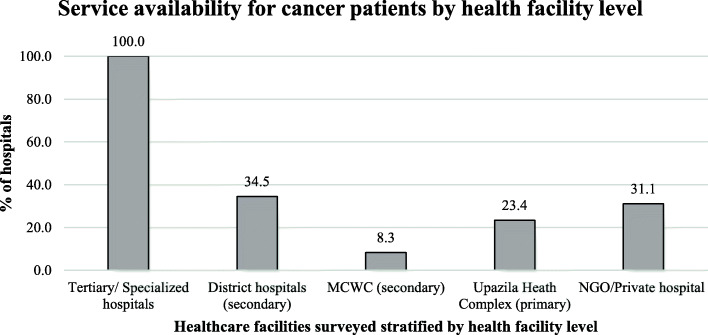
Service availability for cancer patients by health facility level. (surveyed healthcare facilities stratified by level)

Almost three-quarters of the facilities (74%) did not provide any service to cancer patients (Fig. 2). All seven of the tertiary/specialized hospitals provided services for cancer patients, while a few of the UHCs (23.4%) and MCWCs (8.3%) did (Fig. 3). Only about one-third of district hospitals (34.5%) and NGO/private hospitals (31.1%) provided services to cancer patients (Fig. 3).

### Service “readiness” for overall cancer

Frequencies and tracer indicator scores for cancer at each health facility level.

The tracer indicator scores for all 34 cancer tracer indicators at each level of health facility are provided in Table 4.

**Table 4:** Status of tracer indicator scores for all cancer tracer indicators at each level of health facility (*n* = 323)

Tracer Items	Total	Tertiary & Specialized Hospital	District Hospital	MCWC	UHC	NGO/Private Hospital
**Staff and guideline [n (index score in %)]**						
Oncologists	31 (9.6)	7 (100.0)	9 (15.5)	0 (0.0)	1 (0.8)	14 (18.9)
Palliative care specialist	4 (1.2)	3 (42.9)	1 (1.7)	0 (0.0)	0 (0.0)	0 (0.0)
Trained Nurse	9 (2.8)	5 (71.4)	2 (3.4)	0 (0.0)	1 (0.8)	1 (1.4)
Guideline ^a^	41 (12.7)	7 (100.0)	10 (17.2)	3 (5.0)	11 (8.9)	10 (13.5)
Training ^a^	32 (9.9)	7 (100.0)	6 (10.3)	3 (5.0)	13 (10.5)	3 (4.1)
**Equipment [n (index score in %)]**						
Beds available for cancer patients	26 (8.0)	7 (100.0)	7 (12.1)	0 (0.0)	8 (6.5)	4 (5.4)
Speculum ^a^	152 (47.1)	7 (100.0)	36 (62.1)	28 (46.7)	57 (46.0)	24 (32.4)
**Diagnostic facility [n (index score in %)]**						
Acetic Acid ^a^	136 (42.1)	6 (85.7)	36 (62.1)	27 (45.0)	53 (42.7)	14 (18.9)
Sputum for Cytology	80 (24.8)	7 (100.0)	20 (34.5)	1 (1.7)	27 (21.8)	25 (33.8)
Stool occult blood test (OBT)	75 (23.2)	7 (100.0)	14 (24.1)	1 (1.7)	21 (16.9)	32 (43.2)
Cancer antigen 15.3 (CA 15.3)	24 (7.4)	5 (71.4)	3 (5.2)	1 (1.7)	1 (0.8)	14 (18.9)
Cancer antigen 19.9 (CA 19.9)	23 (7.1)	5 (71.4)	3 (5.2)	1 (1.7)	1 (0.8)	13 (17.6)
Carcinoembryonic antigen (CEA)	23 (7.1)	5 (71.4)	3 (5.2)	1 (1.7)	1 (0.8)	13 (17.6)
Cancer antigen 125 (CA125)	23 (7.1)	5 (71.4)	3 (5.2)	1 (1.7)	1 (0.8)	13 (17.6)
Alpha Keto Protein	25 (7.7)	5 (71.4)	3 (5.2)	1 (1.7)	2 (1.6)	14 (18.9)
Endoscopy of upper gastrointestinal tract	43 (13.3)	7 (100.0)	10 (17.2)	0 (0.0)	4 (3.2)	22 (29.7)
Colonoscopy	35 (10.8)	7 (100.0)	8 (13.8)	0 (0.0)	2 (1.6)	18 (24.3)
Pituitary tumor-transforming gene 1-binding factor (PBF)	55 (17.0)	7 (100.0)	14 (24.1)	0 (0.0)	7 (5.6)	27 (36.5)
Bone Marrow Study	21 (6.5)	7 (100.0)	2 (3.4)	0 (0.0)	1 (0.8)	11 (14.9)
Biopsy for Histopathology	35 (10.8)	7 (100.0)	9 (15.5)	0 (0.0)	1 (0.8)	18 (24.3)
Fine Needle Aspiration Cytology (FNAC)	35 (10.8)	6 (85.7)	8 (13.8)	0 (0.0)	1 (0.8)	20 (27.0)
**Medicines [n (index score in %)]**						
Hydrocortisone	150 (46.4)	7 (100.0)	33 (56.9)	18 (30.0)	57 (46.0)	35 (47.3)
Pheniramine	61 (18.9)	5 (71.4)	11 (19.0)	10 (16.7)	17 (13.7)	18 (24.3)
Ondanseron	84 (26.0)	6 (85.7)	20 (34.5)	6 (10.0)	22 (17.7)	30 (40.5)
Haloperidol	40 (12.4)	6 (85.7)	11 (19.0)	3 (5.0)	4 (3.2)	16 (21.6)
Morphin injection	28 (8.7)	6 (85.7)	8 (13.8)	2 (3.3)	4 (3.2)	8 (10.8)
Morphin tablet	23 (7.1)	4 (57.1)	7 (12.1)	1 (1.7)	4 (3.2)	7 (9.5)
Lorazepam	47 (14.6)	5 (71.4)	12 (20.7)	4 (6.7)	10 (8.1)	16 (21.6)
Hyoscine butylebromide	65 (20.1)	7 (100.0)	13 (22.4)	6 (10.0)	21 (16.9)	18 (24.3)
Broad Spectrum Antibiotics	151 (46.7)	7 (100.0)	37 (63.8)	20 (33.3)	55 (44.4)	32 (43.2)
Chemotherapeutic drugs	17 (5.3)	5 (71.4)	2 (3.4)	1 (1.7)	3 (2.4)	6 (8.1)

^a^indicators that are specific for determining service readiness for cervical cancer patients

The tracer indicator scores specific for cervical cancer were 100.0% for guideline, training, and speculum and 85.7% for acetic acid at the tertiary and specialised hospitals. The tracer indicator scores specific for cervical cancer was < 18% for guideline and training, and 62.1% for speculum and acetic acid at the secondary level of health facility, while that at the primary level of health facilities as well as the NGO and private hospitals was < 15 and < 50% respectively.

### Service readiness scores specific for cervical cancer

**Fig. 4:**
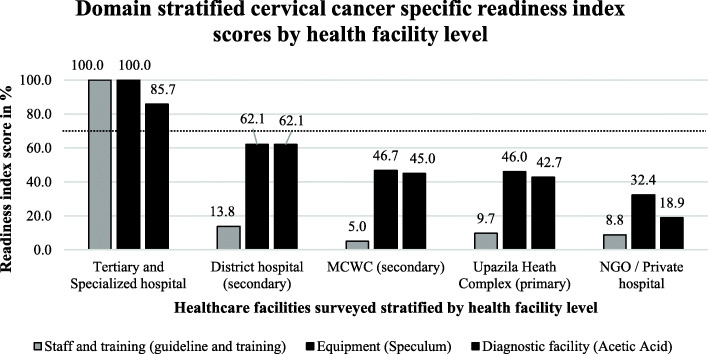
Domain stratified cervical cancer specific readiness index scores by health facility level. (The black dotted line indicates the cut off value 70%, above which a facility is considered to be ‘ready’ to provide services for cervical cancer patients)

Cervical cancer-specific readiness index (CSRI) scores at each health facility level: Fig. 4 shows the domain stratified cervical cancer-specific readiness index (CSRI) scores at each health facility level. All CSRIs at the tertiary and specialized hospitals were above the cutoff score of 70% and therefore “ready” to provide cervical cancer services. The CSRI scores of the staff and training domain (guideline and training tracer indicators), as well as the equipment domain (speculum tracer indicator), were 100%, while that of the diagnostic facility domain (acetic acid tracer indicator) was 85.7%. All CSRIs, among all other facilities, were under the cutoff score of 70%. The CSRI scores were much higher for the equipment domain (62.1, 46.7, 46.0, and 32.4%) and the diagnostic facility domain (62.1, 45.0, 42.7, and 18.9%) than for staff and training domain (13.8, 5.0, 9.7, and 8.8%) among the district hospitals, MCWC, UHC, and private hospitals respectively.

**Fig. 5:**
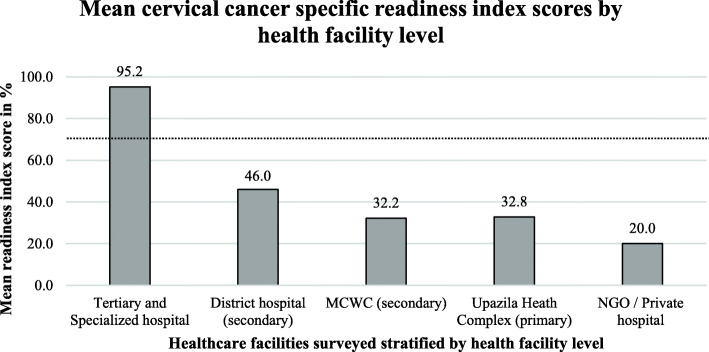
Mean cervical cancer specific readiness index scores by health facility level. (The black dotted line indicates the cut off value 70.0%, above which a facility is considered to be ‘ready’ to provide services for cervical cancer patients)

Mean cervical cancer-specific readiness index (mean CSRI) scores at each health facility level: Fig. 5 shows the mean cancer-specific readiness index (mean CSRI) scores at different types of health facilities. The tertiary level hospitals have a mean CSRI score of 95.2%, above the cutoff value of 70%. The mean CSRI score of the other levels of health facilities was 46% (secondary level district hospitals), 32.8% (primary level health facilities), 32.2% (secondary level MCWCs), and 20.0% (NGOs and private hospitals), which were all below the cutoff value.

## Discussion

This study identified several gaps that require intensive attention by policymakers and implementer to attain the desired targets. During the study, information regarding availability of staff and guidelines, screening equipment, diagnostic facilities and medicines were collected. While issues were relatively good at the tertiary level, more needs to be done at other levels of health facilities. This study also demonstrated that only tertiary-level facilities in Bangladesh are ready to diagnose and treat for cervical cancer patients while only some facilities at the primary and secondary levels have adequate availability and readiness for diagnostic services.

In Ethiopia, a study found that the availability and management of cervical cancer among NCD service providing health facilities was almost negligible, while little over half of the health facilities had trained staff for cervical cancer [3, 22]. Studies in Zambia also showed similar results, which indicates that both service availability and readiness for cervical cancer were low in other developing countries [21]. To the best of the authors’ knowledge, there appears a dearth of existing literature in this field from Asia, even though cervical cancer prevalence is very high in the region. Findings of this study may contribute towards filling the knowledge gap.

Using the SARA tool to assess service availability and readiness for major non-communicable diseases, a study conducted among health facilities in a selected district of Bangladesh found that service availability was less than 40%. The overall readiness score, though variable for different diseases, was close to 70% or below it, indicating that the health services strengthening are warranted to meet the targets set [4]. Similar to what was found for NCDs generally in Bangladesh [4], the service availability and readiness for cervical cancer was also not adequate, as found in this study.

The finding gives the impression that the implementation of the Bangladesh Cancer Control Strategy, which came into effect in 2017 has a long way to go to reach the desired goal by 2022. Less than half of the primary level health facilities and just over half of the secondary level facilities were prepared to diagnose cervical cancer. But in the absence of trained human resources and guidelines, these facilities cannot be brought into action for rendering the desired services. As early detection of cervical cancer is the key to favourable treatment outcome, diagnostic facilities through training and guidelines or standard operating procedure (SOP) need to be strengthened at the primary level. The existing referral mechanism needs to be further streamlined so that the cervical cancer patients can avail treatment at secondary or tertiary level health facilities without delay [23]. The secondary and tertiary level facilities may also have their guidelines or SOPs to diagnose and treat cervical cancer by trained health human resources.

### Strengths and limitations

This study’s strengths included health care facilities from rural to urban under public and/ or private support and explored a large representative sample size. On the other hand, since this was a cross-sectional study and the data collected was only valid for the time when the hospital heads were interviewed. Therefore, no trend or change over time could be witnessed in this study. Also, the study could not include all the sample health facilities due to incomplete questionnaire and absence of proper interviewee. While data on the availability of staff and guidelines, equipment for screening, diagnostic facilities and medicines were collected, information on area specific population density was not collected, which was why bed density, facility density, health worker density, and so forth could not be estimated. Further studies to explore the state of HPV vaccine for the targeted population as per national strategy is needed. While working with the SARA tool, the method has some limitations, specially, for cervical cancer services. The SARA tool allowed data collection on the availability of tracer items relevant to VIA, but not on those relevant for other diagnostic methods such as thermocoagulation and cervical conization. Diagnostic and services for cancer are multidisciplinary requiring specific specialization, an issue not adequately focused on the SARA tool to assess the availability and readiness [24, 25]. As such, this study attempted to find the very basics of availability and readiness, while accepting the limitations of SARA methods.

## Conclusions

The study findings suggest that health facilities in Bangladesh (except for some tertiary level hospitals) lack readiness in cervical cancer management in terms of guidelines on diagnosis and treatment, staff training, and shortage of equipment. Given the high prevalence of cervical cancer in Bangladesh, such cervical cancer management needs to be available at all health facilities levels. If probed, evidence of similar situation may be found in other LMICs too. This study was a basic assessment of service availability and readiness. Further studies can be conducted to collect data on machines for cryotherapy, thermocoagulation and LEEP, capacity to perform cervical conization, hysterectomy and radical hysterectomy, capacity for cancer-related imaging, and capacity for external-beam radiation therapy, brachytherapy, and platinum-based chemoradiation. Alongside this, a well-designed plan to ensure the availability of adequate and competent care services for cervical cancer at public and private sectors following other SARA modules, such as quality of care and safety and management and finance, is needed. It may be appropriate for policymakers to develop standard operating procedure on cervical cancer to guide the care services to achieve the set targets and contribute towards attaining sustainable developmental goals.

## Data Availability

Data is available from the lead author Professor Saidur Rahman Mashreky, PhD. Email. mashreky@ciprb.org

## Abbreviations

RI: Readiness Index
NGO: Non-governmental organization
NCD: Non-communicable disease
LMIC: low- and middle-income country
HPV: Human Papilloma Virus
GOB: Government of Bangladesh
VIA: Visual Inspection of Cervix with Acetic Acid
EPI: Expanded Program on Immunization
PHC: Primary Health Care
NCDC: Non-communicable Disease Control
HSM: Hospital Service Management
MIS: Management Information System
DGHS: Directorate General of Health Services
DGFP: Directorate General of Family Planning
SARA: Service Availability and Readiness Assessment
WHO: World Health Organization
NIPORT: National Institute of Population Research and Training
DH: District hospital
MCWC: Mother and Child Welfare Centre
UHC: Upazila Health Complex
FWC: Family Welfare Centre
CC: Community Clinic
MBBS: Bachelor of Medicine and Bachelor of Surgery
CSRI: Cervical cancer-specific readiness index
SOP: Standard operating procedure
